# Applications of Microfluidics in Liquid Crystal-Based Biosensors

**DOI:** 10.3390/bios11100385

**Published:** 2021-10-12

**Authors:** Jinan Deng, Dandan Han, Jun Yang

**Affiliations:** Key Laboratory of Biorheological Science and Technology, Ministry of Education, Bioengineering College, Chongqing University, Chongqing 400030, China; biojdeng@cqu.edu.cn (J.D.); handandan@cqu.edu.cn (D.H.)

**Keywords:** liquid crystals, microfluidics, biosensors, liquid crystal planar interface, liquid crystal droplets

## Abstract

Liquid crystals (LCs) with stimuli-responsive configuration transition and optical anisotropic properties have attracted enormous interest in the development of simple and label-free biosensors. The combination of microfluidics and the LCs offers great advantages over traditional LC-based biosensors including small sample consumption, fast analysis and low cost. Moreover, microfluidic techniques provide a promising tool to fabricate uniform and reproducible LC-based sensing platforms. In this review, we emphasize the recent development of microfluidics in the fabrication and integration of LC-based biosensors, including LC planar sensing platforms and LC droplets. Fabrication and integration of LC-based planar platforms with microfluidics for biosensing applications are first introduced. The generation and entrapment of monodisperse LC droplets with different microfluidic structures, as well as their applications in the detection of chemical and biological species, are then summarized. Finally, the challenges and future perspectives of the development of LC-based microfluidic biosensors are proposed. This review will promote the understanding of microfluidic techniques in LC-based biosensors and facilitate the development of LC-based microfluidic biosensing devices with high performance.

## 1. Introduction

Liquid crystals (LCs) are materials in a state between the isotropic liquid and the crystalline state, which can not only flow like a fluid, but also exhibit the anisotropic properties of crystalline materials. External stimuli will easily trigger the ordering rearrangement of LCs. With the stimuli-responsive and optically anisotropic properties, LCs have been extensively used in various areas, such as beam steering [1], imaging technology [2], optical filters and switches [3]. The well-known application of LCs is the liquid crystal display. Electrical field is applied to regulate the ordering of LCs between two electrodes to manipulate the light passing through the LCs, and thus the optical appearance.

In the past few decades, as one of the most widely investigated LC phases, the nematic LC phase has been emerging as an optical probe in biosensing fields due to the weak surface anchoring endowed by its long-ranged orientational order but lack of positional order [4,5,6]. It has been shown that the surface anchoring energy of nematic LCs is in the range of 10^−7^ to 10^−3^ mJ/m^2^ [7]. Small perturbations at the surface of LCs can easily change their surface anchoring and thus trigger the ordering transition of LC molecules at the surface. This surface-induced ordering transition of LCs can extend up to 100 μm from the surface into the bulk LCs through their long-range orientational order [8]. In this way, the disturbance at the LC surface can be easily amplified and transduced by the birefringent LCs into optical signals, which can be observed by the naked eye. By the elaborate design and creation of LC-based interfaces, where the LCs re-orientate their ordering to target molecules, specific molecular events could be transduced and amplified into optical signals through polarized light with the birefringent LCs. Compared with traditional biosensing platforms, which usually require complex instruments or fluorophores as reporters, the LC-based sensing platforms provide a simple, rapid, label-free, and cost-effective option for biosensing applications [4,9]. Various LC-based interfaces, such as LC-solid interface, LC-aqueous planar interface, and LC droplet-aqueous interface, have been developed for the detection of biological and chemical species, such as organophosphorus species [10,11,12], endotoxins [13,14,15], cells [16,17], metal ions [18,19,20], protein [21,22,23], nucleic acids [24,25,26], glucose [27,28,29], and bile acids [30,31,32,33]. The ordering of LCs is dictated by the modulation between the elastic energy stored in bulk LCs and the surface anchoring energy of LCs [6]. Compared with LC displays, in which LCs are sealed in LC cells and isolated from the environment, it is challenging to design the LC-based biosensing interface to specifically respond to particular targets. The performance of LC-based biosensors is highly correlated to their surface qualities, geometries and environmental factors. Different chemically treated interfaces would induce a pre-defined orientation of LCs [34,35]. The diversity of functional groups of target molecules and the complex surface interactions between target molecules and the LCs make it difficult to predict the ordering of LCs [36]. It has been revealed that the boundary conditions imposed by the limited volume of microchannels and the curved surface of spherical droplets would strongly affect the formation of defect structures of LCs and thus the arrangement of LCs [37,38,39]. Because LC-based sensing systems should be “open” to allow the analyte to interact with LCs at the LC surface, the variation of ion strength and pH in the local environment would impose an extra torque on the alignment of LCs by forming an electrical double layer and changing the charge of LCs, respectively [40]. Therefore, it requires extra attention and optimization to fabricate stable LC-based biosensors with good quality and reproducibility to ensure the sensing performance. Conventional methods for preparing LC-based biosensors are highly dependent on the manual handling of LCs, which would cause large batch-to-batch variation and greatly hinder the reproducibility. Precise control of the qualities of LC-based sensing platforms remains a challenge in the current development of LC-based biosensors.

**Scheme 1:**
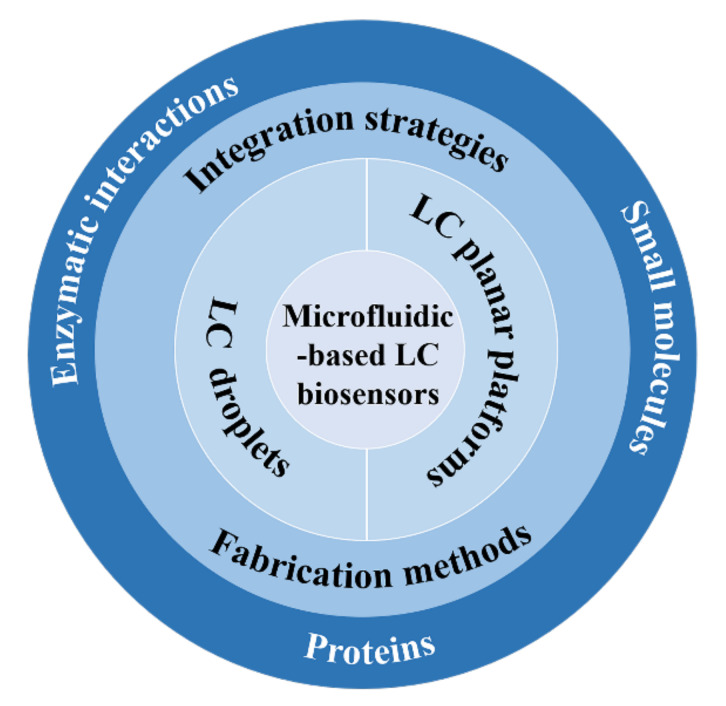
Schematic illustration of the contents in this review.

Microfluidics is a technology that focuses on the precise handling and manipulation of fluids in microscale channels [41]. In past decades, microfluidics has been proven a powerful tool for the synthesis of materials with tunable structures [42,43], precise manipulation of complex fluids [44], and the handling of small quantities of particles in a controlled manner [45]. Moreover, microfluidics provides tremendous advantages over conventional macroscale analysis systems, such as a large reduction of sample consumption and fast interdiffusion [46,47]. Various analytical techniques, such as electrochemical [48], chemiluminescence [49] and optical fibers [50], have been integrated with microfluidic structures to develop miniaturized analysis systems. In recent years, benefiting from the easy integration, highly efficient mass transfer, small sample consumption and portability, microfluidic techniques have been emerging as an efficient tool in the fabrication and integration of LC-based biosensing platforms, offering promising solutions for preparing LC-based biosensors with controlled properties [51,52,53,54,55,56,57]. However, as far as we know, no review is available to summarize different types of LC-based microfluidic biosensors. In this review, we mainly focus on the applications of microfluidic techniques in the fabrication and integration of LC-based platforms for biosensing applications, including LC planar sensing platforms and LC droplets (Scheme 1). We first describe the preparation of LC planar sensing platforms by microfluidic techniques and biosensing applications with the LC planar platform-based microfluidic devices. Then we summarize the methods to generate and capture monodisperse LC droplets using different microfluidic structures and their potential applications in biosensing fields. Finally, the challenges and future perspectives of the applications of microfluidics in LC-based biosensors are discussed.

## 2. Microfluidics in LC Planar Sensing Platforms

**Figure 1:**
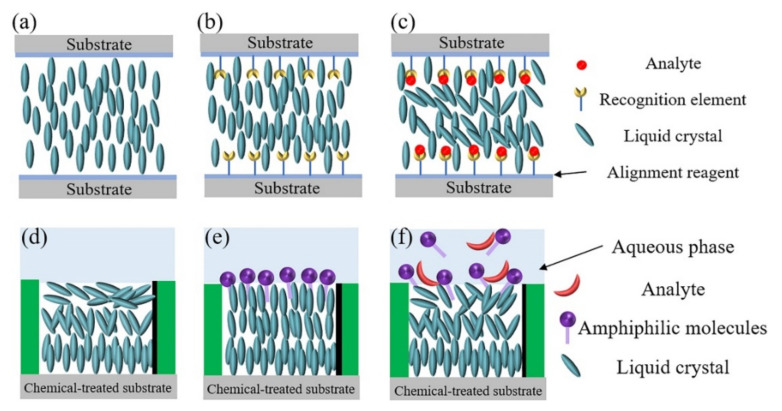
Schematic illustrations of the alignment of LCs on (**a**) alignment reagent-treated substrate, (**b**) recognition element-decorated alignment reagent-treated substrate and (**c**) recognition element-decorated alignment reagent-treated substrate after the addition of analyte. Schematic illustrations of LC-aqueous interface (**d**) without and (**e**) with the assembled amphiphilic molecules. (**f**) The amphiphilic molecules-decorated LC-aqueous interface after the addition of analyte.

There are mainly two types of LC planar sensing platforms, LC-solid interface and LC-aqueous interface, respectively. The LC-solid interface is formed by loading LCs between two solid substrates. The solid substrates are usually glass slides, which are chemically treated with alignment reagents, such as N, N-dimethyl-N-octadecyl-3-aminopropyl trimethoxysilyl chloride (DMOAP) and octyltrichlorosilane (OTS), to predefine a homeotropic alignment of LCs (Figure 1a). In order to detect the target molecules, a specific concentration of recognition element, such as proteins [36,58,59] and nucleic acids [19,60,61], is introduced on the alignment reagent layer of the substrates without disrupting the homeotropic alignment of LCs (Figure 1b), indicating a dark appearance under a polarized optical microscope. The target molecules specifically interacting with the recognition element on the substrate would trigger a tilted alignment of LCs (Figure 1c), exhibiting a bright appearance under the polarized optical microscope and thus can be detected with the naked eye. The LC-aqueous interface is generally created by confining LCs within a transmission electron microscopy (TEM) grid supported on a chemically treated glass substrate to form a planar surface in an aqueous environment (Figure 1d). DMOAP and OTS are usually adopted to treat the glass substrate to induce a homeotropic alignment of LCs near the substrate. The planar LC/aqueous interface is then decorated with amphiphilic molecules, such as phospholipid [62] and surfactant [63], to induce a homeotropic alignment of LCs at the LC-aqueous interface (Figure 1e), suggesting a dark appearance under the polarized optical microscope. The amphiphilic molecules are self-assembled on the LC-aqueous interface with their hydrophobic part extending into the LC phase and their hydrophilic part in contact with the aqueous phase, providing mobile active sites for the targets to interact with due to the fluidity of the LCs. After the addition of analyte, the interactions between the analyte and the amphiphilic molecules could disrupt the packing of the amphiphilic molecules on the LC-aqueous interface and thus induce the ordering transition of LCs (Figure 1f), showing a bright appearance under the polarized optical microscope. By observing the interfacial interaction-induced dark-to-bright optical appearance transitions of the LC planar film under a polarized optical microscope, the detection of analyte could be achieved.

Although various strategies have been employed to construct LC-based planar interfaces, the fabrication of the LC-based interface is still largely dependent on manual operations, and the uniformity and reproducibility of the prepared LC-based interfaces cannot be ensured. For example, during the preparation of the LC-aqueous interface, it is hard to ensure the flatness and uniform thickness of the LC film by manually filling the LCs into the small metallic grid. Even minor differences in the LC-based interface caused by the uncontrollable manual operation could be amplified through LCs, showing obvious appearance differences under a polarized optical microscope and resulting in inconsistent responses [54,64,65]. Microfluidic techniques have provided a promising solution for the fabrication and operation of LC-based interfaces to eliminate the influence of manual preparation and operation on the performance results in terms of their extraordinary ability to precisely control complex fluids [51,66,67]. In this section, the applications of microfluidics in the fabrication and integration of LC-solid and LC-aqueous interfaces for biosensing applications are introduced.

### 2.1. LC-Solid Interface in Microfluidic Channels for Biosensing Applications

#### 2.1.1. Microfluidic Channel Integrated with LC-Solid Interface

**Figure 2:**
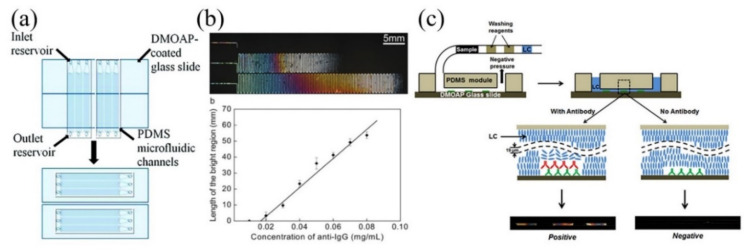
(**a**) Schematic illustrations of the preparation of LC-solid interface with microfluidic techniques for multiplex analysis. Reprinted with permission from ref. [69]. Copyright 2011 American Chemical Society. (**b**) Polarized optical microscopy images of IgG-decorated LC-solid interface-based microfluidic platform after the addition of anti-IgG of (from top) 0.02 mg/mL, 0.05 mg/mL, and 0.08 mg/mL, respectively, and the length of the bright region as a function of the anti-IgG concentration. Reprinted with permission from ref. [68]. Copyright 2009 John Wiley and Sons. (**c**) Schematic illustrations of the working principle of one-step LC-based microfluidic immunoassay. Reprinted with permission from ref. [67]. Copyright 2014 Elsevier.

Generally, there are two ways to construct the LC-solid interface within the microfluidic channels. One way is to apply the recognition element, such as protein or nucleic acid, to the alignment agent treated-glass substrate before binding it with the microchannel pattern. The other way is to bind the treated substrate with the polydimethylsiloxane (PDMS) microchannel first, and then decorate the recognition molecules on the surface of the treated-substrate by injecting them into the microchannel using a micropump. This injection method to immobilize recognition elements on the solid substrate is usually employed to fabricate multiplex analysis platforms [68,69,70]. For example, Zhang and coworkers patterned IgG and biotin-BSA as the recognition elements on a single piece of DMOAP-treated glass substrate by flowing 15 μL of these two solutions into individual PDMS microchannels, followed by incubating for 30 min (Figure 2a) [69]. After incubation, the PDMS microchannel layer was removed and the unabsorbed proteins were washed away with a buffer solution and dried under nitrogen, forming individual IgG and biotin-BSA patterns on the substrate. A new piece of the PDMS microchannel layer was then bonded with the treated glass substrate with the microchannel aligned in the direction perpendicular to the old one (Figure 2a). In that way, different recognition element patterns can be included in a single channel for multiplex analysis. The concentration of the recognition element used to modify the solid substrate should be optimized. A lower concentration of the recognition element would decrease the sensitivity of the platform by reducing the active sites on the substrate, while a higher concentration of the recognition element would induce a tilted alignment of LCs before the addition of analytes, which would reduce the signal contrast ratio, negatively affecting their sensing performance [71]. Moreover, it is worth noting that different from the traditional LC-solid interface within optical cells, in which the anchoring effects of only the bottom and top substrates on the LCs are dominated, the impact of the side walls of the microchannel on the ordering of LCs cannot be neglected [7,67]. Yang and coworkers have investigated the influence of the dimensions of the microchannel on the LC alignment and found that the ordering of LCs inside the microchannel is determined by the aspect ratio of the microchannel [67]. The ordering of LCs is dictated by the top and bottom surfaces of the microchannel with a small aspect ratio, indicating dark in the middle of the microchannel under the polarized optical microscope, while the ordering of LCs is determined by the side walls of the microchannel with a large aspect ratio, showing bright throughout the microchannel under the polarized optical microscope [67]. Thus, it is necessary to optimize the size of the microchannel to obtain an optimal performance before designing the LC-solid interface within the microchannel.

#### 2.1.2. LC-Solid Interface-Based Microfluidic Device for Biosensing Applications

Xue and coworkers demonstrated the feasibility of using IgG pre-coated glass substrate integrated within the microfluidic channel in immunoassay applications [68]. The IgG decorated-substrate induced a homeotropic alignment of the LCs within the microchannel, indicating a dark appearance under the polarized optical microscope. After the addition of anti-IgG of more than 0.02 mg/mL, the LCs show a tilted alignment, indicating a bright appearance under the polarized optical microscope (Figure 2b). The specific binding between the anti-IgG and IgG increased the surface density of proteins adsorbed on the substrate, which would mask the alignment reagent and lower the LC contact angle on the substrate, resulting in the tilted orientation of the LCs [59]. The length of the bright region inside the microchannel shows an increase with the increasing of the anti-IgG concentration. They further found that the length of the bright channel is linearly correlated with the concentration of anti-IgG (Figure 2b) [68]. In order to minimize the influence of manual steps of rinsing on the detection process, Zhu and coworkers developed a one-step LC-based microfluidic immunoassay using a reagent-loaded tubing cartridge [67]. The tubing cartridge was constructed by sequentially loading the sample solution, buffer solution, water and LCs into a tube using a syringe pump and each reagent was separated by air plug (Figure 2c). To perform the test, the cartridge was inserted into the inlet of the microfluidic platform, and then the assay procedures were automatically performed with the aid of a programmed syringe pump. By observing the dark-to-bright appearance transition of LCs under the polarized optical microscope with this one-step microfluidic immunoassay, the anti-rabbit IgG with the detection limit of 1 μg/mL can be obtained. The flow rate of the LCs injected into the microchannel should be carefully chosen. The rapid flow rate of LCs into the microchannel would cause the distortion of LCs and thus the optical appearance of LCs inside the microchannel [70,72].

### 2.2. LC-Aqueous Interface in Microfluidic Channels for Biosensing Applications

#### 2.2.1. Fabrication of LC-Aqueous Interface within Microfluidic Channels

**Figure 3:**
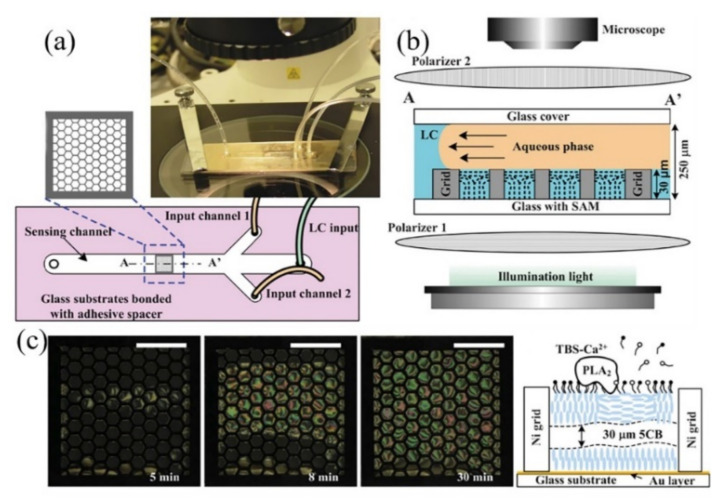
(**a**) Schematic illustration and photograph of microfluidic device for the fabrication of LC-aqueous interface. (**b**) Schematic illustration of the formation of LC-aqueous interface within the microchannel. (**c**) The gradual optical appearance transitions of L-DLPC-decorated LC-aqueous interface within the microchannel after the addition of phospholipase A_2_ in the presence of Ca^2+^ and the related LC ordering transition mechanism. Reprinted with permission from ref. [51]. Copyright 2012 Royal Society of Chemistry.

To fabricate the LC-aqueous interface within the microfluidic channel, a grid microstructure designed to hold the LCs on a glass slide is first fabricated by photolithography technique, followed by coating alignment agents on the surface of the microstructure to pre-define the homeotropic orientation of LCs [66,73]. Compared with traditional preparation methods, in which the TEM grid is usually put on the supporting substrate, the grid microstructure fabricated on the substrate can eliminate the impact of the flow-induced movement of the TEM grid on the detection results [74]. The glass substrate with the grid microstructure is then tightly bonded with a PDMS microchannel to form a microdevice (Figure 3a). The LC-aqueous sensing interface is constructed by sequentially injecting the LCs, deionized water and amphiphilic molecules into the microchannel through a syringe pump. The LCs are loaded into the microstructure by the capillary force and the excess LCs outside the microstructure are removed by the shear force produced by deionized water with a high flow rate (Figure 3b). Finally, specific amphiphilic molecules as the recognition element, are pumped into the channel and assembled on the LC-aqueous interface to form the stimuli-responsive interface. It has been found that the flow behaviors of the fluid inside the microchannel have an influence on the alignment of the LCs [7,75,76]. Jiang and coworkers studied the interactions between the LCs and the aqueous solution within the microchannel during the process of excess LCs removal using the simulation method [51]. They found that the higher flow rate of the aqueous solution can cause higher inner pressure of the device and the appearance of circulations in the flow would damage the device and destroy the integrity of the LC film hosted within the microstructure. Therefore, the parameters of the fluid used to prepare the LC-aqueous interface within the microchannel should be elaborately chosen. Furthermore, in order to simplify the fabrication process of the microwell structure, Lin and coworkers used soft lithography techniques to prepare PDMS-based microwell structure with SU-8 photoresist master mold [77]. After filling the microwell structure with LCs, a microfluidic channel was placed on the substrate to introduce analytes into the sensing surface using a micropump for detection.

#### 2.2.2. LC-Aqueous Interface-Based Microfluidic Device for Biosensing Applications

Many biological events, such as enzymatic reaction, membrane transportation, and ligand-receptor interaction, usually happen on the biological membranes [62]. Construction of interfaces mimicking the fluidic biological membrane is of great importance to the study of the dynamic process of these biological events. It has been revealed that the lipid molecules assembled at the LC-aqueous interface show a similar mobility as in the biological membrane [78]. Moreover, the reorganizations of the molecules at the interface can trigger the re-alignment of LC molecules and thus the molecular events can be transduced and amplified into optical signals through the LCs under the polarized optical microscope. The mobile LC-aqueous interface provides an intriguing label-free platform to study the biological binding event in real-time. By assembling L-α-dilauroyl phosphatidylcholine (L-DLPC) molecules at the mobile LC-aqueous interface constructed inside the microchannel, Jiang and coworkers demonstrated the feasibility of using this platform in the study of enzymatic reactions in real-time and its reusability [51]. The hydrolysis of L-DLPC by the phospholipase A_2_ in the presence of Ca^2+^ leads to the reorganization of the lipid molecules and thus induces the ordering transitions of LCs at the interface, showing a gradual dark-to-bright transition under the polarized optical microscope with time (Figure 3c). In that way, the enzymatic event can be transduced and amplified through the LC film in real-time. In addition, they used ethanol to clean the device to repeat the experiment and found that only less than 5% LC cells were not precisely filled with LCs, indicating a good reusability of this device.

## 3. Microfluidics in LC Droplet-Based Biosensors

**Figure 4:**
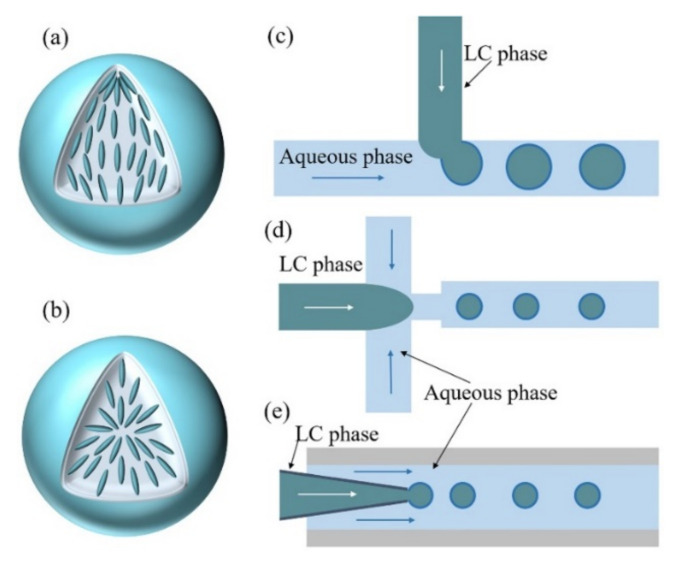
Schematic illustrations of (**a**) bipolar and (**b**) radial configurations of LC droplets. Schematic illustrations of generation of monodisperse LC droplets with (**c**) T-junction, (**d**) flow-focusing and (**e**) co-flow microstructures.

Compared with planar LC films, the preparation of LC droplets does not require substrate and grid structure to support and confine LCs. In addition, LC droplets with a larger surface area to volume ratio and topological defects allow more targets to adsorb on the droplet surface, making them an excellent candidate for sensing applications [14]. There are mainly two configurations of LC droplets. One is a bipolar configuration, where LC molecules align parallel to the surface of the droplet with two point defects at the poles of droplets (Figure 4a). The other one is a radial configuration, where LC molecules orientate perpendicular to the surface of the droplet with a defect at the center of the droplet (Figure 4b). Amphiphilic species are usually used as the recognition elements to functionalize the LC droplet’s surface to induce an initial configuration of LC droplets. The adsorption of target molecules on the surface of LC droplets to interact with the recognition element would trigger the ordering transitions of LCs inside the droplets and thus the configuration transitions of LC droplets. By observing the configuration transitions of LC droplets under a polarized optical microscope, the detection of targets can be achieved.

Different from the planar LC-based interface, in which the ordering of LCs is usually governed by the surface anchoring of LCs, the ordering of LCs inside LC droplets is determined by both the surface anchoring and the droplet size [6,79,80]. The droplet size of LC droplets, dictating the saddle-splay elastic constant, plays a crucial role in the determination of the elastic energy of LC droplets and thus the response behaviors of LC droplets [79,80]. After the addition of 100 pg/mL endotoxin, Abbott and coworkers found that the percentage of LC droplets exhibiting bipolar-to-radial configuration transitions showed a decrease by increasing the droplet size from 2 μm to 10 μm [79]. No configuration transition was observed for droplets with the droplet size larger than 10 μm. However, they found that LC droplets with a too-small droplet size (~0.7 μm) would show a radial configuration without the addition of analyte [80]. They comment that the endotoxin-induced bipolar-to-radial configuration transitions are happening under the conditions where the contributions of the bulk elastic and surface anchoring energies to the free energies of bipolar and radial LC droplets are close to degenerate [79]. Thus, the droplet size should be elaborately designed according to the analyte-induced free energy changes of LC droplets to achieve a better sensing performance. LC droplets prepared by current methods, such as vortex and sonication, exhibit a wide size distribution, which would lead to an inconsistent response [81]. Moreover, although LC droplets are a promising optical probe with high sensitivity, the high mobility of LC droplets makes it hard to study the molecular interactions in real-time. Droplet microfluidics, which focuses on the generation and manipulation of droplets within microfluidic channels in a controllable way, offers versatile platforms for producing and manipulating LC droplets [47,82,83]. In this section, the applications of microfluidics in the fabrication and entrapment of monodisperse LC droplets for biosensing applications are introduced.

### 3.1. Microfluidic Fabrication of LC Droplets for Biosensing Applications

#### 3.1.1. Fabrication Methods for Monodisperse LC Droplets

**Figure 5:**
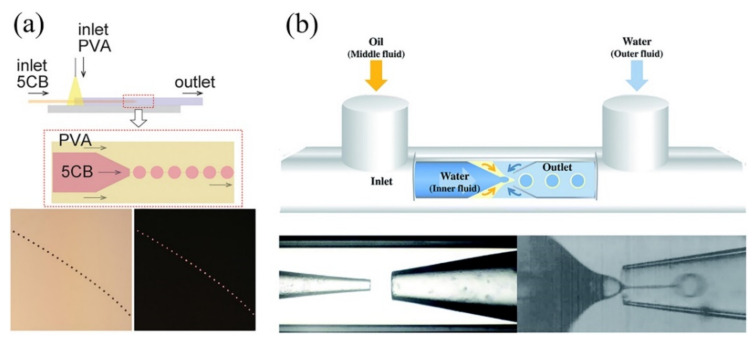
(**a**) LC necklace structure prepared by co-flow microstructure. Reprinted with permission from ref. [93]. Copyright 2020 American Chemical Society. (**b**) LC double emulsion droplets produced by the combination of co-flow and flow-focusing geometries. Reprinted with permission from ref. [89]. Copyright 2016 Royal Society of Chemistry.

Typically, there are mainly three types of microfluidic structures employed to prepare monodisperse LC droplets, the co-flow structure, flow-focusing structure, and T-junction structure [84]. The co-flow microstructure is usually manufactured with a glass capillary by inserting a tapered cylindrical glass capillary into a square one, which is chemically resistant and allows a wide range of materials for the generation of droplets [85]. The T-junction and flow-focusing microstructures are usually fabricated by polydimethylsiloxane (PDMS) using the soft lithography method, which is suitable for large-scale production. The inner phase or dispersed phase is LCs, and the outer phase or the continuous phase is an aqueous solution containing amphiphilic molecules. In the T-junction microstructure, the LC phase flows perpendicular to the aqueous phase (Figure 4c). In the flow-focusing structure, the aqueous phase flows from two side channels to cut the LC phase in the middle channel (Figure 4d). In co-flow geometry, the LC phase and the aqueous phase flow in the same direction (Figure 4e). Syringe pumps are usually used to control the flow rates of the two phases. When the two immiscible phases meet at the junction, the LC phase will become unstable and break up into droplets due to Rayleigh instability with amphiphilic molecules adsorbing on the surface of LC droplets. The accumulation of amphiphilic molecules on the surface of LC droplets can stabilize the generated LC droplets in aqueous solution to prevent the droplet coalescence. This breakup process is a sophisticated phenomenon, which is dictated by various parameters, such as viscous force, interfacial tension, channel geometries, and flow conditions [86,87]. Additionally, the channel wall surface should be treated to be effectively wetted by the continuous phase to ensure the stable formation of LC droplets [88]. Moreover, by manipulation of the fluids within the microchannels and the design of microfluidic structures, some interesting and complex structures of LC droplets can be fabricated [89,90,91,92,93,94]. For example, by controlling the viscosity and flow rate of the poly (vinyl alcohol) (PVA) continuous phase inside a co-flow geometry, Takanaka and coworkers prepared a necklace-like structure, in which PVA-stabilized LC droplets connected by thin tethers made up of PVA and LC in one step (Figure 5a) [93]. By combining co-flow and flow-focusing geometries using glass capillaries, where tapered cylindrical glass capillaries were assembled into a square capillary and were firmly and coaxially aligned, Park and coworkers produced LC double emulsion droplets with LC phase as the middle layer (Figure 5b) [89]. The aqueous solution containing surfactant was injected from the left inner capillary and the LC phase was introduced in the same direction through the space between the left inner and the square capillaries. The LCs broke up the inner aqueous phase at the tip of the left inner capillary to form W/O emulsions due to capillary instability and then met the continuous aqueous phase containing polyelectrolyte flowing in the opposite direction, resulting in W/O/W double emulsion droplets with LCs as the middle layer.

#### 3.1.2. Biosensing Applications with Monodisperse LC Droplets

**Figure 6:**
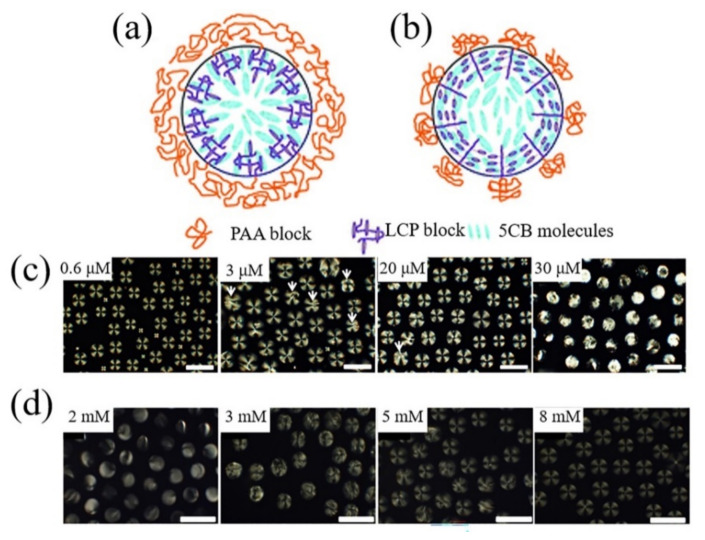
Configurations of PAA-b-LCP-decorated LC droplet at (**a**) higher pH and (**b**) lower pH. Reprinted with permission from ref. [52]. Copyright 2011 Royal Society of Chemistry. (**c**) The polarized optical microscopy images of GOx-coated LC droplets with different glucose concentrations. Reprinted with permission from ref. [27]. Copyright 2013 American Chemical Society. (**d**) The polarized optical microscopy images of urease-coated LC droplets with different urea concentrations. Reprinted with permission from ref. [95]. Copyright 2014 Elsevier. The scale bar is 100 μm.

By elaborate decoration of the LC droplet surface with amphiphilic molecules as the recognition element, the detections of pH [52,89], glucose [27], urea [95], bile acids [96] and avidin-biotin binding [97] have been reported. Park and coworkers synthesized a pH sensitive copolymer, poly (acrylicacid-b-4-cynobiphenyl-4- oxyundecylacrylate) (PAA-b-LCP), to prepare LC droplet-based pH sensor in a flow-focusing microstructure [52]. The weak polyelectrolyte of PAA block would adopt different conformations in different pH solutions. At higher pH, the swelling of PAA coils induces an oblate configuration of LCP block and triggers LCs to align perpendicular to the surface of LC droplet, indicating a radial configuration (Figure 6a). At a lower pH, the PAA chains become compact, allowing LCP blocks to extend deeper into the droplet and induce a parallel alignment of LCs inside the droplet, suggesting a bipolar configuration (Figure 6b). The radial-to-bipolar configuration transition of LC droplets started at a pH of 5 and completely change to bipolar at pH of 4. Using this pH-dependent response of PAA-b-LCP-coated LC droplets, they further immobilized glucose oxidase (GOx) and urease on the PAA-b-LCP copolymer to decorate LC droplets and explored their feasibility in the detection of glucose [27] and urea [95], respectively. The local pH changes caused by the GOx-catalyzed oxidation of glucose and urease-catalyzed hydrolysis of urea can induce the swelling and shrinkage of PAA chains of PAA-b-LCP, respectively, and thus trigger the ordering transitions of LCs in the droplets. The GOx-coated LC droplets show a bipolar configuration at a pH of 7 under the polarized optical microscope, while the H^+^ released by the oxidation of glucose by GOx would lead to the decrease of the local pH, causing the shrinkage of the PAA block thus triggering the radial-to-bipolar configuration transition of LC droplets (Figure 6c). With GOx-PAA-b-LCP-decorated LC droplets, the detection limit of 30 μM for glucose can be obtained. In order to realize the detection of urea, the initial pH was kept at 6 to retain the bipolar configuration of urease-PAA-b-LCP-coated LC droplets. After the addition of urea with a concentration of more than 3 mM, the generated OH^−^ from the hydrolysis of urea by urease increased the local pH of the solution, leading to the shrinkage of PAA coils, thus inducing the bipolar-to-radial configuration transition of LC droplets (Figure 6d).

### 3.2. Confinement of LC Droplet within Microfluidic Channel for Real-Time Detection

Uniform properties of monodisperse LC droplets produced by microfluidic techniques allow an analysis with high reproducibility. However, the high mobility of LC droplets in an aqueous solution makes it hard to study the molecular interactions in real-time. Therefore, the development of microstructures to trap and separate LC droplets into individual chambers is of great interest to the study of reaction dynamics happening on the surface of LC droplets at single droplet level [47]. Static and dynamic strategies are usually used to confine the mobile LC droplets, forming LC droplet-microarrays for biosensing applications.

#### 3.2.1. LC Droplet-Based Static Microarrays

In static microarrays, LC droplets are immobilized on a chemically treated-solid substrate to induce a homeotropic alignment of LCs near the substrate. Monodisperse LC droplets are generated by the inkjet printing technique and the droplet size is controlled by printing different times. The surface of the LC droplet is decorated with amphiphilic molecules as the recognition element and then the LC droplet-immobilized substrate was integrated into a microfluidic channel as the sensing platform for real-time detection applications [54]. It has been found that the surface of smaller droplets is more like a planar film, showing a dark appearance under the polarized optical microscope by adsorption of hexadecyltrimethylammonium bromide (CTAB) on the surface, while larger droplets with a curved surface show a bright appearance after the adsorption of CTAB on the surface [54]. Thus, it is better to optimize the droplet size in the preparation of LC droplet-based static microarray to get a better sensing performance. It is worth noting that the flow condition inside the microfluidic channel should be cautiously handled considering the facile detachment of LC droplets from the substrate by the shear force exerted by the fluid within the microfluidic channel [98].

**Figure 7:**
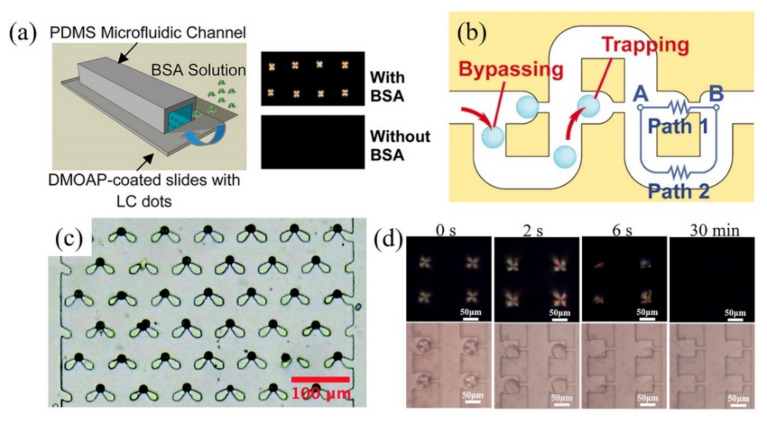
(**a**) LC droplet-based static microarray for the detection of BSA. Reprinted with permission from ref. [54]. Copyright 2013 American Chemical Society. (**b**) The schematic diagram of the trap mechanism using by-pass microfluidic channel. Reprinted with permission from ref. [100]. Copyright 2007 National Academy of Science of the USA. (**c**) Optical microscopy image of LC droplets trapped in a swallowtail shaped microstructure. Reprinted with permission from ref. [57]. Copyright 2019 Royal Society of Chemistry. (**d**) Polarizing optical microscopy and corresponding bright-field microscopy images of LC droplet-based microarray after the introduction of 20 μM cholic acid at a constant flow rate of 3 μL/h. Reprinted with permission from Reference [55]. Copyright 2020 Elsevier.

Yang and coworkers used inkjet printing techniques to immobilize LC droplets on a DMOAP treated-solid substrate with CTAB as the stabilizer and further integrated the LC droplet-immobilized substrate with a microfluidic channel to form a static microarray for BSA detection [54]. After the addition of BSA, the interaction between BSA and CTAB disrupted the packing of CTAB on the surface of LC droplets and thus induced the dark-to-bright optical appearance transition of LC droplets (Figure 7a). By dividing the LC droplet-based microarray into 37 sections and counting the number of bright sections after the addition of BSA, the BSA concentration could be quantitatively detected.

#### 3.2.2. LC Droplet-Based Dynamic Microarrays

Compared with stationary solid support in the static microarray, LC droplets in the dynamic microarray are captured individually in a trapping chamber surrounded by an aqueous environment [55,57]. The inlet size and the outlet size of the trapping chamber are larger and smaller than the droplet size, respectively. A passive pressure is usually applied to direct the droplets into the traps. After the entrapment of droplets into the traps, the outlet of the trap is blocked by the trapped droplet, preventing the second droplet from moving into the trap, and the other droplets are then redirected into other empty traps [99,100,101]. In order to improve the trapping efficiency, bypass-channel trapping microstructure was designed to capture the droplets sequentially in a short time (Figure 7b) [100]. The bypass-channel trapping microstructure usually includes a main loop channel and straight channels with trapping chambers superimposed on the straight path. The geometry of the channel plays an important role in manipulating the resistance between the main loop channel and the straight route. The straight channel should be designed with a lower flow resistance than that along the main loop channel in order to allow the droplets to enter the empty chamber [100]. After the trapping chamber is occupied by the droplet, the flow resistance in this chamber is dramatically increased, and the subsequent droplet will bypass the occupied chamber and be directed into the next chamber with lower flow resistance. In this way, the droplets can be sequentially filled into the empty traps.

Bao and coworkers designed a swallowtail-shaped microstructure as the trapping chamber and a tree-shape network microstructure as the concentration gradient generator to detect Smp43, a cationic antimicrobial peptides (AMPs) [57]. The lipid-coated LC droplets prepared by flow-focusing microstructure were captured by the microarray and then different concentrations of Smp43 generated by the concentration gradient generator were simultaneously introduced into six microarrays. The interactions between lipid molecules and AMPs with the concentration of higher than 6 μM can disrupt the packing of lipid molecules on the surface of LC droplets, triggering the radial-to-bipolar configuration transitions of LC droplets. By observing the configuration transition process of LC droplets under the polarized optical microscope, the interactions between the AMPs and lipid molecules can be monitored in real-time. It is worth noting that the flow-induced ordering change of LCs inside the LC droplet should be taken into consideration for the on-chip analysis. It has been shown that the relative motion of LC droplets to the aqueous flow inside the microchannel would induce a flow inside the LC droplet, which can trigger the configuration change of LC droplets [102]. With sodium dodecyl sulfate (SDS) and PVA as the stabilizers, Han and coworkers prepared monodisperse LC droplets using flow-focusing microstructure and captured them into a bypass-channel microarray for real-time detection of bile acids [55]. They found that the defect point of radial LC droplets showed a shift from the center in an aqueous environment with a flow rate of 200 μL/h, while the defect point returned back after the flow rate lowered to 3 μL/h. They attributed this phenomenon to the shear force-induced deviation of SDS from the surface of LC droplets exerted by the flowing fluid. They further performed the real-time detection of bile acid by continuously injecting specific concentrations of cholic acid into the microarray with the flow rate of 3 μL/h and found that the radial LC droplets gradually shrank and finally disappeared with time (Figure 7d). The shrinkage of LC droplets is due to the removal of SDS from the droplet surface by the cholic acid and the LC phase is then partitioned into the flowing continuous phase [99]. It is worth noting that the performance of LC droplet-based biosensors is strongly dependent on the droplet density. By altering the LC droplet density within the microfluidic channel for the detection of bile acids, Fang and coworkers found that the detection limits for cholic acid and deoxycholic acid decrease with the decrease of LC droplet density [56].

## 4. Conclusions

Microfluidic techniques provide a versatile tool for the fabrication of uniform LC-based sensing platforms with high reproducibility. In this review, LC planar platforms and LC droplets fabricated by microfluidic techniques and their applications in biosensing fields were discussed. By modification of the surface of LC sensing platforms with amphiphilic molecules as the recognition element with microfluidic techniques, a wide range of chemical and biological species can be detected (Table 1). Moreover, the combination of microfluidics and LC-based sensing platforms offers enormous advantages over traditional LC-based sensors, including small sample consumption, rapid analysis, easy integration and automatic operation with high reliability, showing a great potential in point-of-care testing (POCT) applications. Although many efforts have been made to study the applications of microfluidics in LC-based sensors, great challenges still remain. For example, the complex interactions between analytes and recognition elements at the LC interface under a flowing environment within the microchannel make the prediction of LC ordering difficult. It is necessary to elucidate the underlying mechanisms of ordering transitions of LCs in the LC-based microfluidic sensing platform, which is essential to guide the design of the LC-based sensing interface inside the microchannel with excellent performance.

Microfluidic techniques offer an efficient tool to eliminate the interference of manual operation on the sensing performance of LC-based sensors. However, the potential response of LC-based sensing platforms to the interfering biological and chemical species still exists. Seeking methods to improve the selectivity and sensitivity of the LC-based microfluidic sensors is of great significance. Aptamers with high stability, structure tunability and high selectivity and affinity for targets may be a good choice as the recognition element to prepare LC-based microfluidic sensing platforms with a superior performance [23,107,108,109]. In addition, using machine learning techniques for data analysis may provide another future direction to enhance the selectivity of LC-based microfluidic sensors. Compared with the adoption of naked eyes to distinguish the differences in the optical appearance of LCs, the machine learning techniques can uncover valuable information underlying the microscopy images of LCs, providing a more accurate detection result [13,110,111,112]. To date, the LC-based biosensors are still studied in laboratories using a polarized optical microscope to observe the detection results, and real breakthroughs are still expected to develop a portable, easy-to-use LC-based sensing device for commercial applications.

## Figures and Tables

**Table 1:** Summary of LC-based microfluidic biosensors.

Sensing Structure	Type of LC	Surface Modification of LC	Target	Linear Range	Detection Limit	Ref.
LC-solid interface	5CB	IgG	Anti-IgG	0.02–0.08 mg/mL	0.02 mg/mL	[68]
	5CB	Biotin-BSA	Cy3 antibiotin	Not reported	5 μg/mL	[69]
	5CB	Rabbit IgG	Anti-rabbit IgG	Not reported	1 μg/mL	[67]
	E44	BSA	Anti-BSA	Not reported	1 μg/mL	[71]
	E7 doped with dichroic dye	BSA	Anti-BSA	Not reported	1 μg/mL	[103]
LC-aqueous interface	5CB	L-DLPC	Phospholipase	Not reported	100 nM	[51]
	5CB doped with HBA	None	Creatinine	50–250 μM	50 μM	[77]
Mobile LC droplets	5CB	PAA-b-LCP-glucose oxidase	Glucose	Not reported	0.03 mM	[27]
	5CB	PNIPAM-b-LCP/SDS	BSA Lysozyme Hemoglobin Chymotrypsinogen	Not reported	0.95 μM 1.1 μM 0.12 μM 0.07 μM	[104]
	5CB	SDS/QP4VP	BSA Hemoglobin	Not reported	0.005 wt% 0.03 wt%	[105]
	5CB	PAA-b-LCP-urease	Urea	Not reported	3 mM	[95]
	5CB	PAA-b-LCP-avidin	Avidin	Not reported	0.5 μg/mL	[97]
	MLC-2132 doped with CB15	PAA-b-LCP-glucose oxidase	Glucose	Not reported	0.5 μM	[106]
		PAA-b-LCP-cholesterol oxidase	Cholesterol	Not reported	2.5 μM	
	E7 doped with R5011	PVA/SDS	Cholic acid Deoxycholic acid	Not reported	1 μM 0.5 μM	[96]
LC droplet microarray	5CB	CTAB	BSA	4–40 μg/mL	4 μg/mL	[54]
	E7	Lipid mixture	Smp43	Not reported	4.8 μM at 2 h 3.6 μM at 4 h	[57]
	5CB	PVA/SDS	Cholic acid	10–30 μM	10 μM	[55]
			Deoxycholic acid	1–10 μM	1 μM	

## Data Availability

Not Applicable.
